# Molecular and Cellular Mechanisms Underlying Domoic Acid-Induced Neurotoxicity and Therapeutic Drugs: A Comprehensive Review

**DOI:** 10.3390/ijms27020867

**Published:** 2026-01-15

**Authors:** Ruoyu Jiang, Zeyu Fan, Xinhao Li, Jiaping Yang, Mingjuan Sun, Binghua Jiao, Lianghua Wang

**Affiliations:** Department of Biochemistry and Molecular Biology, College of Basic Medical Sciences, Naval Medical University, Shanghai 200433, China; smmujry@smmu.edu.cn (R.J.); zeyufan@smmu.edu.cn (Z.F.); hydlxh@smmu.edu.cn (X.L.); smmu_shenghuayjp@163.com (J.Y.); sunmj@smmu.edu.cn (M.S.)

**Keywords:** domoic acid, marine biological toxins, amnesic shellfish poisoning, GluRs, reactive oxygen species, calcium overload

## Abstract

Domoic acid (DA) is a neurotoxic terpenoid compound produced by certain marine algae. It accumulates through the food web and poses a significant threat to humans and animals by selectively targeting hippocampal neurons, leading to neuronal degeneration, necrosis, and subsequent memory impairment. The primary mechanism involves its potent agonism at glutamate receptors, which induces excessive calcium influx, resulting in excitotoxic cell swelling and death. Recent studies have further elucidated the critical role of downstream oxidative stress and other pathogenic factors in DA-induced neurotoxicity. These insights into its multifaceted mechanism have paved the way for novel therapeutic strategies, highlighting promising directions for future treatment development.

## 1. Introduction

Red-tide diatoms such as *Pseudo-nitzschia multiseries* can synthesize the amino acid-derived toxin domoic acid (DA), a potent neurotoxin responsible for amnesic shellfish poisoning (ASP) [1]. DA targets hippocampal neurons, leading to their degeneration and necrosis, which ultimately results in memory impairment. Both DA and its isomers accumulate through marine food webs, concentrating in tissues of primary and secondary consumers, including numerous commercially harvested species. Consequently, diverse marine food chains are affected, with documented impacts on sea lions, whales, and seabirds [2]. Through its accumulation in the food chain, DA can be concentrated in different marine organisms, and ingestion by humans and non-human primates can cause gastrointestinal effects, while slightly higher doses can cause neurological symptoms, seizures, memory impairments, and limbic system degradation [3]. The first confirmed human poisoning event occurred in 1987 in eastern Canada, when over 100 individuals fell ill after consuming contaminated blue mussels (*Mytilus edulis*) from Prince Edward Island, resulting in at least three fatalities [4,5]. This incident prompted extensive research into the ecology, physiology, and distribution of toxigenic *Pseudo-nitzschia* species.

Chemically, DA is a crystalline, water-soluble neurotoxic amino acid (C_15_H_21_NO_6_; MW 311.33) with at least nine known geometric isomers, each exhibiting lower toxicity than DA itself [6]. The compound is a white, solid powder soluble in water (8 mg/mL) and methanol (0.6 mg/mL), with stability highly dependent on pH. DA is photosensitive: diluted solutions exposed to ultraviolet light undergo reversible photoisomerization to isodomoic acids D, E, and F, as well as irreversible decarboxylation [7]. Structurally analogous to the excitatory neurotransmitter glutamate, DA acts as a potent agonist at kainate, NMDA (N-Methyl-D-Aspartate), and AMPA (α-Amino-3-hydroxy-5-methyl-4-isoxazolepropionic acid) receptors [8,9]. Due to its higher binding affinity, DA competitively displaces glutamate, leading to receptor overactivation, sustained calcium (Ca^2+^) influx, neuronal swelling, and ultimately cell death. The specific vulnerability of hippocampal neurons underlies the characteristic memory deficits that define ASP [10].

Currently, no specific antidote exists for DA poisoning. Standard care relies on symptomatic management, such as using antiepileptic drugs (e.g., benzodiazepines and phenobarbital) to control seizures [11]. Experimental drugs that interfere with the activation of AMPA/KA or NMDA receptors may hold some promise for the future [12]. In this review, we present the damage mechanisms of DA and its effects on animals, humans, and the environment. We also summarize research findings regarding the roles of neuroinflammation and oxidative stress in brain dysfunction caused by DA and emphasize novel and promising therapeutic strategies for treating DA damage through new targets involving neuroinflammation and oxidative stress pathways.

## 2. Distribution of the Genus *Pseudo-nitzschia* and DA Production

### 2.1. Toxic Species

DA is produced by specific organisms in two distantly related branches of algae: planktonic diatoms and red macroalgae [13]. It was originally isolated in the 1950s by Daigo and his collaborators from the red alga *Chondria armata*, hence the name domoic acid [13,14]. Diatoms that can produce DA include *Pseudo-nitzschia multiseries*, *Pseudo-nitzschia multist riata*, *Pseudo-nitzschia delicatissima*, and *Pseudo-nitzschia pungens* [15]. To date, 54 species of *Pseudo-nitzschia* have been identified worldwide, of which 26 have been identified as producing DA that is lethal to humans, birds, and marine mammals [16,17]. Among them, *Pseudo-nitzschia australis* was the main type of diatom that caused the most severe DA poisoning incident in history, namely the Prince Edward Island incident in Canada in 1987.

The toxic diatom is not a localized phenomenon. It has been reported in numerous temperate and frigid seas around the world, including the Pacific Rim regions (the west coast of North America, Chile, New Zealand, Japan, and the coastal areas of China), the North Atlantic (northern Europe, the east coast of Canada), and the Southern Ocean [18,19,20]. A recent study based on metabolomics data has confirmed that the distribution of toxin-producing strains extends globally from polar regions to coastal and open marine environments, covering a temperature range from −1.81 °C to 31.2 °C [21]. However, there are several areas of the world, mainly tropical and polar regions, where the presence of *Pseudo-nitzschia* has not been assessed, meaning that there may be many more species that remain undiscovered [21].

### 2.2. Influencing Factors of DA Production

It has been speculated that all species of *Pseudo-nitzschia* may produce DA if given the right conditions [4]. The factors affecting DA production include biological factors and abiotic factors. Research shows that bacterial community composition is correlated with whether *Pseudo-nitzschia* species produce domoic acid [22]. Sison-Mangus MP et al. confirmed that *Pseudo-nitzschia* is associated with a unique and diverse group of bacteria; these bacteria regulate their algae-killing activity by producing enzymes, while the type of algae secretions of the host may in turn regulate the symbiotic or parasitic relationship between the bacteria and the diatom host [23].

Production of DA is affected by light and temperature, as these factors affect *Pseudo-nitzschia* cell growth and enzyme activity [19]. *Pseudo-nitzschia* exhibits increased growth under warm conditions [24]; therefore, at higher temperatures and higher radiation intensities (the light intensity is about 120 μmol photons m^−2^s^−1^), DA production tends to be higher [25]. In addition, different lighting schemes can also affect the results. Various nutrients, such as nitrogen, phosphorus, and silicon, have significant effects on DA production in *Pseudo-nitzschia*. Recent studies have confirmed that silicates and phosphates limit the triggering of DA production [15], which is validated in the transcriptional activity model established by Brunson JK et al., showing that phosphate restriction and CO_2_ rise can stimulate DA production [26]. Trace metals, especially iron and copper, also have a certain influence on DA production. Iron can promote DA biosynthesis by affecting redox reactions in metabolic processes such as respiration and photosynthesis [27]. The effect of copper on DA production is still controversial, and the results vary by species and growth stage [17]. DA can form chelates with iron and copper; thus, *Pseudo-nitzschia* may increase metal absorption or reduce metal toxicity by increasing the production of DA. However, Lelong et al. found that there was no change in the intracellular and extracellular DA content in *Pseudo-nitzschia* species under copper stress [28]. Bates et al. reviewed in detail the physicochemical, biological, nutritional, and trace metal factors influencing DA production or cellular physiology in different *Pseudo-nitzschia* species [4].

## 3. Molecular and Cellular Mechanisms

The greatest threat that DA poses to living organisms is its toxicity to the nervous system. The structural similarity between DA and the endogenous neurotransmitter glutamate forms the basis of its toxicity. After activating glutamate receptors, DA triggers the influx of ions, which in turn activates a series of downstream cascade reactions [29,30].

### 3.1. Glutamate Receptor Excitotoxicity

Domoic acid is an excitatory neurotoxin that is structurally similar to glutamic acid and kainic acid and can directly bind to glutamic acid receptors in vivo. Glutamate receptors (GluRs) are divided into two categories: The first category comprises ionotropic glutamate receptors, including α amino-3-hydroxy-5-methyl-4-isozazole propionic acid (AMPA) receptor, kainic acid (KA) receptor, and N-methyl-D-aspartate (NMDA) receptor, which are coupled with ion channels to form receptor–channel complexes, mediating fast signal transmission [31,32]. The other category belongs to metabotropic glutamate receptors (mGluRs), which are coupled to G proteins in the membrane and are activated to act through a signal transduction system composed of G-protein efferent enzymes and second messengers in the brain, resulting in slower physiological responses [33]. Glutamate (Glu) is the major excitatory neurotransmitter in the mammalian central nervous system. During neural signaling, glutamate is released from the presynaptic membrane into the synaptic gap when a neuron sends a message to a neighboring neuron and activates downstream signaling pathways by binding to specific types of GluR. When DA is present, because its molecular structure is very similar to that of the main excitatory neurotransmitter, glutamate, in the brain [34]. Therefore, it can specifically and with high affinity bind to and activate ionotropic glutamate receptors in the central nervous system.

**Figure 1:**
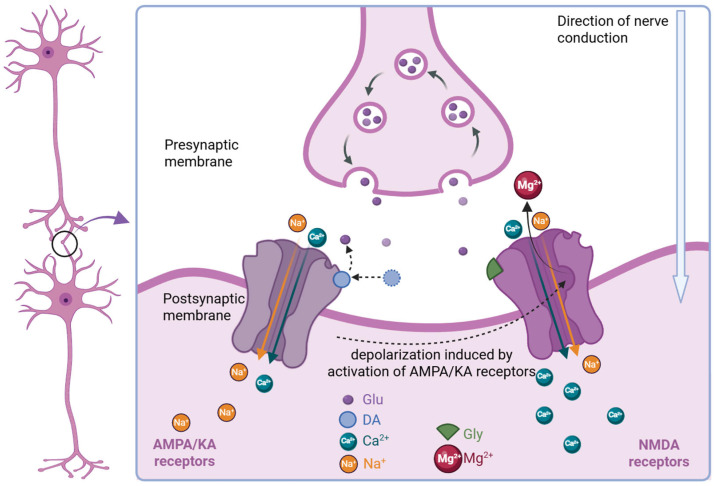
DA competitively binds to the glutamate receptor and triggers the influx of calcium ions. For AMPA/KA receptors (left), DA competes with glutamate and tightly binds to the binding site, keeping the ion channel open, allowing Na^+^ and Ca^2+^ to enter, thereby causing depolarization of the dendritic membrane and continuous transmission of nerve impulses. In the resting state, the ion channel of NMDA receptors (right) is blocked by Mg^2+^. After DA binds to the receptor, the AMPA/KA receptors are activated, glycine participates in binding, and the postsynaptic membrane depolarizes, jointly promoting the release of magnesium ions, thereby activating NMDA receptors and causing a large influx of calcium ions (created in BioRender. Jiang, R. (2026) https://BioRender.com/pqywons).

Under normal circumstances, glutamate transiently binds to the binding sites of AMPA/KA receptors on the postsynaptic membrane [35], and is taken back into the presynaptic axon through endocytosis. When DA is present, it competes with glutamate and forms a tight binding with the binding site. Binding of DA to AMPA receptors exerts a non-desensitizing effect on receptor kinetics, thereby promoting sustained Na^+^ influx into the postsynaptic dendrite; depending on the editing state of the AMPA receptor, this may also permit the entry of a small amount of Ca^2+^. The resulting inward current leads to dendritic membrane depolarization and facilitates continuous nerve impulse transmission. In contrast, DA acts differently at KA receptors, where it induces a desensitizing response. Although stimulation of either AMPA or KA receptors can effectively depolarize the postsynaptic neuron, the depolarization mediated by AMPA receptors is longer-lasting due to their non-desensitizing properties [36]. Compared to AMPA/KA receptors, DA has a relatively lower affinity for NMDA receptors [35]. In the resting state, NMDA receptor ion channels are blocked by Mg^2+^. At low concentrations, DA first activates non-NMDA receptors, leading to depolarization of the neuronal membrane, which relieves the voltage-dependent Mg^2+^ block of NMDA receptor channels [37]. The activation of AMPA/KA receptors by DA triggers a massive influx of Ca^2+^, and this influx of Ca^2+^ and disruption of homeostatic balance are closely associated with cell death [37,38] (Figure 1).

### 3.2. Calcium Overload and Oxidative Stress

A study based on a network toxicology strategy identified the calcium signaling pathway as one of the key pathways affected by DA and enriched several potential targets related to DA exposure-induced memory loss and neurotoxicity, including Rap1, TNF, estrogen, VEGF, MAPK, and AKT [39]. These targets have also been verified in previous studies [40,41,42]. Calcium ions, as important second messengers, trigger a series of fatal molecular mechanisms, ultimately leading to cell death. During this process, excessive Ca^2+^ in the cytoplasm is taken up by mitochondria in large quantities in an attempt to maintain cytoplasmic calcium homeostasis. The high calcium load induces continuous opening of the mitochondrial permeability transition pore, leading to mitochondrial swelling, outer membrane rupture, and the release of pro-apoptotic factors such as cytochrome C [43,44]. At the same time, the electrochemical gradient across the inner mitochondrial membrane is disrupted, and collapse of the membrane potential uncouples oxidative phosphorylation process, severely hindering ATP synthesis and causing cellular energy depletion [43]. The damaged mitochondrial electron transport chain generates large amounts of superoxide and other reactive oxygen species (ROSs), exacerbating oxidative stress [45,46]. Excessive reactive oxygen species attack lipids and trigger lipid peroxidation reactions, while also attacking proteins and DNA, thereby causing damage to the structure of the cell membrane [47]. For instance, ROS plays a crucial role in the pathogenesis of cognitive deficits induced by DA by inducing the activation of the stress-activated protein kinase/c-jun N-terminal kinase (SAPK/JNK) pathway [48].

**Figure 2:**
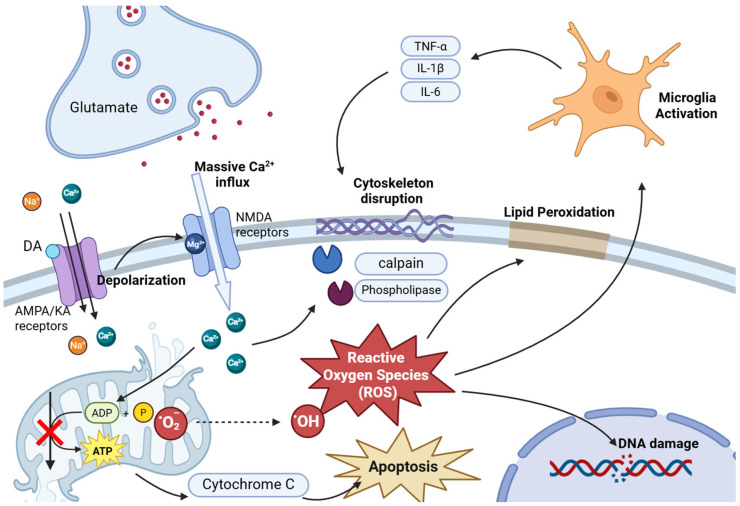
Calcium overload and oxidative stress. Elevated intracellular calcium induces mitochondrial swelling and outer membrane rupture. This process impairs ATP synthesis and promotes the release of apoptotic factors such as cytochrome C, along with substantial amounts of superoxide and other reactive oxygen species (ROSs), thereby exacerbating oxidative stress. Excessive ROS attack lipids, proteins, and DNA, leading to structural damage to the cell membrane. Concurrently, activated microglia release large quantities of pro-inflammatory cytokines, intensifying neuroinflammation and excitotoxicity (created in BioRender. Jiang, R. (2026) https://BioRender.com/rqw4b7p).

Due calcium ion overload, a variety of calcium-dependent degrading enzymes are activated, including calpains, phospholipases, and endonucleases [49]. Activation of these enzymes causes damage to proteins such as the cytoskeleton and further disrupts the integrity of the cell membrane. Furthermore, the destruction and damage of the neuronal structure activate immune cells in the brain, including microglia and astrocytes [50,51]. The response of microglia can even occur at doses that do not cause any change in cell morphology [50]. The activated microglia release a large amount of pro-inflammatory cytokines, such as tumor necrosis factor-α (TNF-α), interleukin-1β (IL-1β), and interleukin-6 (IL-6) [52]. These factors recruit more immune cells and directly act on neurons, exacerbating neuroinflammation and excitotoxicity and forming another vicious cycle [53]. The underlying mechanisms of DA-induced damage, including inflammatory and oxidative stress processes leading to apoptotic and necrotic cell death [53,54] (Figure 2).

### 3.3. Cell Death

In excitotoxicity induced by domoic acid, cell death is not a single pattern but a complex process in which multiple modes interweave and influence one another [55]. When calcium overload causes the mitochondrial permeability transition pore to remain continuously open, the mitochondria first undergo swelling, which confirms the occurrence of necrotic apoptosis [8,30]. After mitochondrial damage, the apoptotic pathway regulated by Bcl-2 family proteins and the caspase cascade reaction are triggered. These findings have been confirmed in the study through results such as caspase-3 activation, DNA damage, and changes in the ratio of Bax/Bcl-2 [48,56,57,58,59]. Among them, the damage caused by DA to DNA is not in the form of double-strand breaks, but rather in the form of single-strand breaks or alkaline susceptible bases [56]. Moreover, the degree of damage shows significant dose and time dependence [56]. Furthermore, caspase-1-mediated pro-inflammatory programmed necrosis triggered by DA indicates that cell death mediated by it may be related to pyroptosis [60]. The mode of cell death induced by DA in the same cell type varies with the concentration of DA. For instance, when DA acts on mouse cerebellar granule neurons (CGNs), the mode of cell death depends on its concentration: when the chondroitin concentration was above 0.1 μM, the cells were predominantly damaged by necrosis, whereas exposure to lower concentrations of chondroitin (≤0.1 μM) is characterized by apoptosis [61].

It has been demonstrated that DA induces cellular MDA upregulation and ROS production and interferes with GSH metabolism, resulting in cellular oxidative stress and even death [62,63], which has similarities with the characteristics of cellular iron death. Our study found that DA-induced reductions in microglia cell activity could be partially reversed by iron death inhibitors; thus, it is reasonable to assume that iron death is involved in DA-mediated cytotoxicity.

## 4. Harm to People, Animals, and Environment

DA is a naturally occurring algal toxin that causes amnesic shellfish poisoning (ASP) in humans. Produced by diatoms such as *Pseudo-nitzschia* and *Nitzschia*, DA enters the food web when ingested by copepods and other organisms, accumulating at higher trophic levels. For instance, DA is found in all tissues of razor clams and in the internal organs of mussels and fish [64,65]. Recent studies further indicate that DA can affect pelagic seabirds through contaminated fish, thereby contributing to broader marine environmental pollution [66]. Consequently, the primary exposure risk for humans and marine wildlife stems from consuming filter-feeding organisms such as shellfish and fish that have accumulated DA. This risk is exacerbated by global warming and the increasing frequency of harmful algal blooms. *Pseudo-nitzschia* spp. are among the dominant phytoplankton groups, and high concentrations of DA have been detected in scallops (*Chamys nobilis*) during bloom events, as well as in nearly all phytoplankton samples [67]. Given the projected rise in the intensity and frequency of toxic *Pseudo-nitzschia* blooms worldwide, cases of DA-induced ASP are expected to increase [68].

Consistent evidence indicates that DA exposure induces neurotoxicity- and epilepsy-related symptoms in mice, with older mice showing greater susceptibility to acute neurotoxic effects [69]. Rats exposed to DA exhibited prolonged epileptic status and increased aggression [70]. In non-human primates, acute DA exposure led to marked excitotoxicity, dyskinesia, and tremors, while long-term low-level exposure triggered adaptive responses in white matter and myelin that mitigated subtle neurological impacts [71,72]. Among wildlife, most data derive from California sea lions, in which acute symptoms—including seizures, ataxia, head and muscle tremors, reduced responsiveness, and coma—either subsided within seven days of DA clearance or progress to chronic epilepsy or death. The chronic condition is characterized by recurrent seizures, hippocampal atrophy, and often behavioral changes and spatial memory deficits [73]. In humans, ASP manifests clinically with gastrointestinal distress, confusion, disorientation, seizures, short-term memory loss, and, in severe cases, death [74,75]. Based on the analysis conducted by Pulido OM, we can consider the 1987 Canadian mussel poisoning incident as a typical case of acute neurotoxicity caused by domoic acid [75]. It mainly activates the necrotic cell death pathway within excitotoxicity [75]. However, in subacute or chronic low-dose domoic acid exposure models, apoptotic pathways may play a more significant role [75]. During the acute phase, patients may experience headaches, epilepsy, hemiplegia, ophthalmoplegia, and altered arousal. Months later, some develop significant anterograde memory impairment. Thus, DA poisoning is initially marked by widespread neurological dysfunction, which may later evolve into chronic memory deficits and motor neuropathy or axonopathy [76].

More alarmingly, DA has been detected in infant plasma and amniotic fluid at delivery, with infant concentrations positively correlating with maternal plasma and amniotic fluid levels, indicating that DA exhibits high toxicity toward newborns and even embryos [77,78].

### 4.1. Pathological Change After DA Exposure

Pathological damage after DA exposure varies depending on the species, exposure situations, and length of recovery after exposure. In the central nervous system, histopathologic evidence suggests that the hippocampus and other brain regions are specific target sites that are highly sensitive to DA toxicity [59]. Damage to limbic structures in the brain after DA exposure is the most common and serious outcome, with severe damage observed in the entire conus cell layer and dentate granule cell layer of the hippocampal areas CA1, CA3, and CA4 [79]. In addition, the amygdala, pyriform cortex, thalamus, septum, olfactory nodes, and retina are also damaged [79]. Acute DA brain injury is characterized by neurodegenerative changes mainly in limbic system structures, including neuronal atrophy, cytoplasmic vacuolization, cellular detachment, edema, microvacuolization of neuronal cells, and cytoplasmic edema of astrocytes [80]. Chronic DA injury is characterized by pathological manifestations of chronic lesions in the hippocampus and parahippocampal gyrus, with severe neuronal loss leading to atrophy of the brain parenchyma, and the presence of astrocytes and oligodendrocytes gliocytosis [75,80]. Chronic lesions were most severe in the dentate gyrus and hippocampal CA3 region, although other regions were also affected [80].

### 4.2. Cytotoxicity of Nervous System Cells

Studies have demonstrated the toxic and associated effects of DA on various cell types. While the cytotoxicity of DA in non-target cells, such as HepG2 hepatocytes, erythrocytes, and Caco-2 enterocytes, has been detailed elsewhere [81], this section focuses primarily on its impact on cells of the nervous system.

In neurons, domoic acid exposure has been shown to significantly reduce the number of dopaminergic neurons and decrease the expression of neuronal nuclear antigen [63]. Morphologically, high concentrations of DA shorten axonal length [82]. Notably, even at sub-cytotoxic concentrations, DA can markedly alter neuronal spiking patterns and overall activity, suggesting impairment of neurological function prior to the onset of clinical symptoms [83]. Regarding neural stem cells, both cytotoxic and non-cytotoxic concentrations of DA inhibit the differentiation of rat neural stem cells (rNSCs) into astrocytes, neurons, and oligodendrocytes [82]. Furthermore, DA directly affects glial cells: it induces changes in astrocyte gene expression—including early response genes, chemokines, tyrosine kinases (Trks), and apoptotic genes—consistent with a role in neurodegenerative processes [84]. Microglia become activated, upregulating MHC II antigens and CR3 receptors to function as antigen-presenting cells, while also enhancing their phagocytic activity [51]. At the ultrastructural level, DA exposure leads to observable alterations such as markedly enlarged perinuclear spaces and nuclei in hippocampal astrocytes, swollen synaptic morphology, and accumulation of intermediate filament bundles in the cytoplasm [84].

It is noteworthy that DA exhibits a marked cellular selectivity in its neurotoxicity. Studies indicate that GABAergic interneurons are far more sensitive to DA than glutamatergic principal neurons [85]. This heightened vulnerability is primarily attributable to the specific receptor repertoire expressed by interneurons, which is enriched in calcium-permeable AMPA receptors and KA receptor subtypes (e.g., GluK2) that possess exceptionally high affinity for DA [85]. This receptor profile renders them particularly vulnerable to unregulated calcium overload triggered by DA [85].

### 4.3. Developmental Neurotoxicity

The developmental neurotoxicity of DA has garnered increasing attention. Studies indicate that even at sub-cytotoxic exposure levels, DA can disrupt neuronal activity, implying that early-life exposure may persistently alter neural function without overt clinical symptoms. Notably, the developing nervous system exhibits far greater sensitivity to DA than that of adults. Animal studies confirm that DA concentrations one to two orders of magnitude lower than the toxic dose for adults can induce significant neurotoxicity in developing organisms—an effect that is markedly attenuated or absent in mature animals [86].

DA can cross the placental barrier and directly threaten fetal neurodevelopment, particularly during critical windows of brain growth and differentiation [87]. DA reaching the placenta can enter fetal brain tissue and has been linked to persistent motor abnormalities and cognitive deficits in offspring, although severe congenital malformations are generally not observed [86]. Comparative toxicological evidence further suggests that DA-induced damage to the central nervous system may be lasting and could even progress with age [86].

In zebrafish embryo models, DA exposure induces a range of developmental defects. By seven days post-fertilization, embryos display behavioral abnormalities such as reduced fear response and altered movement patterns [88]. Hatched larvae show high mortality and characteristic signs of developmental toxicity, including pericardial edema, yolk sac edema, spinal curvature, and abnormal cardiac morphology [88]. These phenotypes correlate with dysregulated expression of genes involved in tissue integrity and myelination [89,90].

In mammalian models, neonatal DA exposure in mice leads to emotion-related behavioral abnormalities that become more pronounced with aging. One proposed mechanism is the rapid postnatal downregulation of high taurine levels in the immature brain. Taurine normally counteracts glutamate neurotoxicity by reducing intracellular free calcium; its decline during the first week after birth may render developing neurons especially vulnerable to DA-induced excitotoxicity [91].

### 4.4. Harm to Non-Target Organs

Following ingestion, DA can be detected in the gastrointestinal tract, liver, bile, and kidneys [92]. The majority of the absorbed toxin is subsequently excreted via renal and biliary routes, a process that serves to limit its accumulation in sensitive neural tissues [92,93]. Notably, the kidneys are responsible for the primary elimination of DA [93], which leads to its preferential accumulation within this organ, particularly in the proximal tubules. This renal accumulation contributes to toxicity, as DA is believed to promote the influx of sodium and calcium ions into tubular and endothelial cells, resulting in cellular swelling and damage [49,94]. Doses as low as 0.1 mg/kg have been shown to elevate biomarkers indicative of kidney injury [49,94].

In addition to renal effects, DA exerts a direct impact on the gastrointestinal tract. Abdominal cramps and diarrhea are common early symptoms of poisoning [95,96]. The mechanism is linked to DA binding to glutamate receptors on the gastrointestinal mucosa after absorption, which triggers calcium overload. This Ca^2+^ dysregulation can disrupt cellular signaling and cause excitotoxicity, potentially leading to direct stimulation of enteric neurons and smooth muscle, thereby inducing intestinal cramping, abdominal pain, and altered peristalsis [95].

### 4.5. Relationship with Alzheimer’s Disease

A current hypothesis suggests that long-term, low-dose dietary intake of DA may be associated with an increased risk of certain neurodegenerative diseases, such as Alzheimer’s disease, as the chronic, low-level excitotoxicity and neuroinflammation it induces may resemble the pathological processes underlying these disorders [97]. Mechanistically, rapid activation of NMDAR receptors leads to a sharp overload of Ca^2+^ in neurons, triggering acute excitotoxicity within hours to days [98,99]. In contrast, persistent modest elevations in intracellular calcium coupled with deteriorating signaling pathways can provoke chronic excitotoxicity. These alterations are capable of disrupting synapses and neural networks in brain regions critical for cognitive function [98]. Subsequently, increased activity of β- and δ-secretases promotes the deposition of amyloid proteins [100,101]. Thus, chronic excitotoxicity may contribute to the delayed onset of Alzheimer’s disease through both secretase-dependent and secretase-independent pathways [100].

Beyond Alzheimer’s disease, glutamate receptors play a central role in the central nervous system and are implicated in a range of neurological disorders. They also contribute significantly to the pathogenesis of other neurodegenerative diseases, including Parkinson’s disease and amyotrophic lateral sclerosis [102,103].

## 5. Protection, Detection, and Treatment

### 5.1. Protection

Currently, protection against DA primarily relies on non-specific personal protective equipment (PPE), such as protective clothing and gloves, to prevent cuts and puncture wounds when handling toxic shellfish. Risk assessment for DA poisoning can be based on factors such as dose–response relationships and exposure levels; however, while the severity of adverse effects in humans correlates with the ingested dose of DA, this correlation is difficult to predict accurately and is subject to uncertainty. This is largely because estimates often rely on patient recall of consumed shellfish quantities post-poisoning, and DA concentrations are typically extrapolated from mussel samples collected from affected areas after an outbreak [96].

Certain parts of aquatic products—such as the feet of clams, crab viscera, and the hepatopancreas—can contain relatively high concentrations of DA. Consumption of large quantities of seafood from contaminated waters may therefore lead to chronic low-level DA exposure [96]. Monitoring DA levels in water and seafood is an effective measure to avoid dietary intake, and many countries and agencies establish shellfish harvesting and consumption limits based on detected DA concentrations [104]. For instance, Canada was the first to implement a regulatory mechanism that suspends shellfish harvesting when monitoring reveals DA concentrations of 20 mg/kg or higher in shellfish tissue [105]. This limit was derived from DA levels measured in mussels during the Prince Edward Island poisoning incident (approximately 200 mg/kg in mussel tissue) and applies a safety factor of 1270 [71]. When hyponatremia occurs in the body, the physiological Na^+^/Ca^2+^ exchange mechanism becomes impaired [34]. Under such conditions, exposure to domoic acid can enhance its necrotic neurotoxicity due to the hyponatremic state [34]. The U.S. Food and Drug Administration (FDA) has set an action level of 20 ppm in shellfish tissue, equivalent to about 0.075–0.1 mg/kg body weight per day, to protect consumers from toxic exposure [106]. This limit is considered well below the approximate no-observed-adverse-effect level in mice and is thus deemed protective against acute human poisoning [106].

Additionally, during shellfish processing, attention should be given to factors such as environmental conditions, inter-organ variability in DA concentrations, and potential cross-contamination.

### 5.2. Detection

Commonly used methods for detecting domoic acid (DA) include bioassays, enzyme-linked immunosorbent assays (ELISA), high-performance liquid chromatography (HPLC), capillary electrophoresis, and biosensor-based techniques [17,107,108]. Among these, ELISA is widely employed as a screening and quantitative tool due to its operational simplicity. Recently, Kim JH et al. developed a direct competitive ELISA (dc-ELISA) platform that offers an innovative alternative to conventional antibody-based assays [109]. This system utilizes affinity peptides identified through phage display and chemically synthesized with biotin labeling, thereby enhancing both the stability and sensitivity of DA detection [109].

For diagnosing individuals suspected of amnesic shellfish poisoning (ASP), the rapid metabolism of DA in the body and potential delays in collecting fluid samples can result in toxin levels falling below the detection limit. In such cases, neuropathological examination, electromyography (EMG), electroencephalography (EEG), and magnetic resonance imaging (MRI) serve as valuable diagnostic tools [79].

Beyond direct DA detection, predicting harmful algal blooms offers a proactive approach to preventing DA outbreaks. For example, RPA-LFD (Recombinase Polymerase Amplification Combined with Lateral Flow Dipstick Assay) enables rapid and highly sensitive detection of *Pseudo-nitzschia multiseries* by targeting the internal transcribed spacer region of its nuclear ribosomal DNA [110]. This early-warning system can forecast DA levels up to one week in advance, playing a crucial role in mitigating the adverse effects of algal blooms on the environment, aquaculture, and public health [68,110].

### 5.3. Treatment

#### 5.3.1. Treatment for Relieving Epilepsy

Here, we focus on the clinical management of human domoic acid (DA) exposure. Current treatment for DA-induced seizures in amnesic shellfish poisoning (ASP) includes a range of interventions, such as diazepam and therapeutic hypothermia [105]. Benzodiazepines are commonly required for symptom control, with some reports indicating that lorazepam may be more effective than diazepam [79]. Studies in animal models offer further insight: Gulland et al. observed that high-dose phenobarbital more effectively controlled seizures in DA-poisoned California sea lions and may help prevent further hippocampal damage resulting from seizure-related hypoxia [11]. Breakthrough seizures in these animals were managed with combinations of lorazepam, midazolam, and/or diazepam along with phenobarbital [11]. In human clinical practice, standard anticonvulsants have sometimes failed to prevent seizures in severe ASP cases; however, withholding anticonvulsant therapy may lead to clinical deterioration [76]. Olney and colleagues have proposed the use of thiobarbiturates and procyclidine, either alone or in combination, and suggest that calcium channel blockers may also merit consideration in future therapeutic strategies [111].

#### 5.3.2. Glutamate Receptor Blocking Drugs

Glutamate receptor antagonists, particularly those targeting AMPA and KA receptors, such as CNQX and NBQX, have been instrumental in elucidating the neurotoxic mechanisms of domoic acid (DA) in laboratory studies and have demonstrated potential neuroprotective properties [34,63]. Research indicates that both NMDA receptor antagonists (e.g., MK 801) and AMPA receptor antagonists (e.g., NBQX) can attenuate DA-induced neurotoxicity in cellular and animal models [63,112]. In hippocampal studies, MK 801 was found to significantly enhance the neuroprotective effect of NBQX against DA in the CA1 and dentate gyrus subregions, although it showed no notable effect in the CA3 region [112]. Using midbrain primary cultures, Radad et al. demonstrated that the AMPA/KA receptor antagonist NBQX completely blocked DA neurotoxicity at low-DA concentrations and preserved a substantial number of dopaminergic neurons even at high concentrations. In contrast, the NMDA receptor antagonist MK 801 only showed significant protection under low-DA conditions [9,63]. The temporal dynamics of excitotoxicity also inform potential treatment strategies. Prehn et al. observed that MK 801 protects hippocampal neurons during the acute phase of glutamate-induced injury, whereas NBQX remains effective in later stages [113]. This suggests that when NMDA receptor blockade becomes less effective, AMPA receptor inhibition may offer a viable intervention window.

Despite promising preclinical results, the clinical application of glutamate receptor antagonists for DA poisoning remains limited, primarily due to their side effect profiles and the lack of supporting clinical trial data.

#### 5.3.3. Antioxidant Drugs

There is growing evidence that reactive oxygen species (ROSs) production and oxidative stress- induced mitochondrial dysfunction represent key mechanisms underlying DA-mediated cognitive deficits. Therefore, therapeutic strategies aimed at improving mitochondrial function and enhancing oxidative stress defense may be beneficial in DA poisoning [48,81]. For instance, Wang et al. reported that quercetin alleviated DA-induced cognitive impairment in mice by activating AMPK to attenuate mitochondrial dysfunction and stimulating the Nrf2 pathway to bolster antioxidant defenses, leading to improved performance in novel object recognition and Morris water maze tasks [114]. Similarly, Chen et al. demonstrated that Urolithin A (Uro A) mitigates DA-induced damage by promoting mitochondrial biogenesis via estrogen receptor α, reducing oxidative stress through suppression of p47phox/gp91phox, and inhibiting ER stress-mediated apoptosis, ultimately improving synaptic function and memory [115]. Purple sweet potato pigment has also been shown to act via stimulation of estrogen receptors [116]. Furthermore, Wu et al. found that ursolic acid (UA) counteracts DA-induced mitochondrial dysfunction by modulating the PI3K/Akt signaling pathway and FoxO1, a key regulator of mitochondrial homeostasis [117]. These agents collectively reduce mitochondrial oxidative stress triggered by DA and may represent promising candidates for preventing or treating excitotoxicity and cognitive deficits in other brain disorders. Additional compounds, such as extracts from Terminalia arjuna bark, melatonin, and ginseng ginsenosides, have also shown neuroprotective effects, largely attributed to their antioxidant properties [30,38,118]. Here we have summarized the main mechanisms, target sites and current application situation of antioxidant drugs mentioned in the current research (Table 1) [30,61,114,115,116,117,118].

Beyond antioxidant approaches, other compounds act downstream of ROS generation to alleviate DA-induced neural injury [117]. For example, carbachol inhibits DA-triggered activation of JNK and p38 kinases, reduces mitochondrial translocation of the pro-apoptotic protein Bax, and suppresses caspase 3 activation [42]. Troxerutin reverses DA-associated memory impairment in mice by inhibiting the MEK/ERK1/2/C/EBPβ pathway [119]. Interestingly, nanomedicine offers dual potential—not only as sensitive biomarkers for early DA exposure but also as therapeutic agents [120]. Metal nanoparticles (NPs) can induce the expression of mammalian metallothioneins (MTs) and low-molecular-weight cysteine-rich proteins [120]. DA-induced mitochondrial degeneration promotes MT production and the formation of Charnoly bodies. MTs enhance mitochondrial bioenergetics by upregulating NADH ubiquinone oxidoreductase, a rate-limiting enzyme in oxidative phosphorylation, thereby exerting antioxidant effects [120]. These findings suggest that nanotechnology holds broad promise not only for DA detection but also for therapeutic intervention in DA poisoning [121].

## 6. Special Applications of DA

Most studies on learning and memory impairments and related neurological disorders rely on experimental models, many of which are induced by neurotoxins such as DA [122]. First, DA serves as a pharmacological tool for modeling excitotoxicity and related neurological conditions—including epilepsy and Alzheimer’s disease—as previously outlined [103]. Notably, autism spectrum disorder (ASD) shares substantial biological and behavioral overlap with the detrimental effects of DA exposure, encompassing repetitive behaviors, social interaction deficits, and seizure-like activity. Anatomical and functional parallels also exist: the aberrant brain connectivity observed following DA exposure resembles the dysregulated network patterns characteristic of ASD. These similarities support the use of DA-exposed mice as a model system for studying this neurodevelopmental disorder [123,124]. Furthermore, DA has been shown to elicit schizophrenia-like phenotypes, reinforcing the role of glutamate receptor dysregulation in the pathogenesis of schizophrenia and related clinical symptoms [125].

## 7. Conclusions

Domoic acid (DA) is a potent neurotoxin produced by certain marine diatoms and red algae, with widespread implications for marine ecosystems, wildlife, and human health. Its structural resemblance to glutamate enables it to act as a strong agonist at ionotropic glutamate receptors (AMPA, KA, and NMDA), leading to excessive calcium influx, mitochondrial dysfunction, oxidative stress, and ultimately neuronal death. These molecular events underlie the characteristic hippocampal damage and memory impairment observed in amnesic shellfish poisoning (ASP).

Beyond acute excitotoxicity, DA exposure triggers sustained neuroinflammation, activates microglia and astrocytes, and promotes multiple forms of cell death, including apoptosis, necrosis, and potentially ferroptosis. The toxin also exhibits developmental neurotoxicity, crossing the placental barrier and affecting fetal brain development, with long-lasting cognitive and motor deficits. Moreover, emerging evidence links chronic low-level DA exposure to neurodegenerative processes resembling those in Alzheimer’s disease, highlighting its potential role in late-onset neurological disorders.

Current management of DA poisoning remains supportive, focusing on seizure control with antiepileptic drugs. While glutamate receptor antagonists and antioxidant agents show promise in preclinical studies, their clinical translation is limited by side effects and a lack of human trials. Advances in detection methods, such as improved ELISA platforms and molecular forecasting of algal blooms, offer proactive strategies to mitigate exposure risks.

Among the promising therapeutic agents mentioned in Section 5.3.3, we can observe that natural compounds are notably present. These compounds possess multi-bioactivities, including antioxidant and anti-inflammatory effects, and signaling pathway modulation, along with relatively favorable safety profiles, making them attractive sources for lead compound libraries. For future treatment of domoic acid poisoning, we can develop multifunctional agents capable of synergistically enhancing intrinsic cellular protective mechanisms (such as mitochondrial biogenesis) while inhibiting key damage pathways (such as ER stress-mediated apoptosis). Moreover, such molecules should exhibit strong blood–brain barrier penetration and selectively act on neuroprotective targets (e.g., Erα and Nrf2).

## Figures and Tables

**Table 1:** The main mechanisms, target sites, and current application situation of antioxidant drugs for DA treatment.

Drug/Compound	Core Therapeutic Mechanism	Key Targets and Pathways	Reference	Animal Behavior Test Verified
Quercetin	Attenuates mitochondrial dysfunction and oxidative stress	Activates AMPK/PGC-1α and Nrf2 antioxidant pathways	Wang et al., 2018 [114]	Yes
Urolithin A	Promotes mitochondrial biogenesis; reduces ER stress and apoptosis	Acts via ERα to enhance NRF-1/TFAM; suppresses NOX expression	Chen et al., 2025 [115]	Yes
Ursolic Acid (UA)	Improve mitochondrial function	Activates PI3K/Akt; inhibits FoxO1 nuclear translocation	Wu et al., 2013 [117]	Yes
Purple Sweet Potato Color	Enhances mitochondrial biogenesis	Acts through ERα, upregulating NRF-1, TFAM, and mitochondrial complexes	Lu et al., 2012 [116]	Yes
Terminalia Arjuna extract	Reduces oxidative stress and prevents apoptosis	Modulates ROS/NO levels; enhances antioxidant enzymes (catalase, GR); preserves mitochondrial membrane potential	Ramya et al., 2022 [118]	No
Melatonin	Reduces oxidative/nitrosative stress via direct free-radical scavenging	Inhibits pro-apoptotic proteins (e.g., Bax, Caspase-3)	Reiter et al., 2010 [30]; Giordano et al., 2007 [61]	Yes
Troxerutin	Inhibits C/EBPβ-mediated inflammation and oxidative stress	Suppresses PKC/K-ras/Raf/MEK/ERK/C/EBPβ pathway and reduces TNF-α, NF-κB, and NADPH oxidase activity	Lu et al., 2012 [116]	Yes

## Data Availability

No new data were created or analyzed in this study. Data sharing does not apply to this article.
